# 37 The Impact of the Intersectionality of Sex and Race on Body Image in Burn Survivors

**DOI:** 10.1093/jbcr/irae036.037

**Published:** 2024-04-17

**Authors:** Andrew Humbert, Kara McMullen, Alyssa M Bamer, Caitlin M Orton, Kimberly Roaten, Jeffrey C Schneider, Kyra Solis-Beach, Nhi-Ha Trinh, Haig A Yenikomshian, Dagmar Amtmann

**Affiliations:** University of Washington, Seattle, WA; University of Washington, Golden, Colorado; UW Medicine Regional Burn Center, Harborview Medical Center, Seattle, WA; UT Southwestern/Parkland Health, Dallas, Texas; Spaulding Rehabilitation Hospital/Harvard Medical School, Boston, MA; UT Southwestern Medical Center, Dallas, Texas; Massachuett General Hospital, Boston, Massachusetts; University of Southern California, Los Angeles, CA; University of Washington, Seattle, WA; University of Washington, Golden, Colorado; UW Medicine Regional Burn Center, Harborview Medical Center, Seattle, WA; UT Southwestern/Parkland Health, Dallas, Texas; Spaulding Rehabilitation Hospital/Harvard Medical School, Boston, MA; UT Southwestern Medical Center, Dallas, Texas; Massachuett General Hospital, Boston, Massachusetts; University of Southern California, Los Angeles, CA; University of Washington, Seattle, WA; University of Washington, Golden, Colorado; UW Medicine Regional Burn Center, Harborview Medical Center, Seattle, WA; UT Southwestern/Parkland Health, Dallas, Texas; Spaulding Rehabilitation Hospital/Harvard Medical School, Boston, MA; UT Southwestern Medical Center, Dallas, Texas; Massachuett General Hospital, Boston, Massachusetts; University of Southern California, Los Angeles, CA; University of Washington, Seattle, WA; University of Washington, Golden, Colorado; UW Medicine Regional Burn Center, Harborview Medical Center, Seattle, WA; UT Southwestern/Parkland Health, Dallas, Texas; Spaulding Rehabilitation Hospital/Harvard Medical School, Boston, MA; UT Southwestern Medical Center, Dallas, Texas; Massachuett General Hospital, Boston, Massachusetts; University of Southern California, Los Angeles, CA; University of Washington, Seattle, WA; University of Washington, Golden, Colorado; UW Medicine Regional Burn Center, Harborview Medical Center, Seattle, WA; UT Southwestern/Parkland Health, Dallas, Texas; Spaulding Rehabilitation Hospital/Harvard Medical School, Boston, MA; UT Southwestern Medical Center, Dallas, Texas; Massachuett General Hospital, Boston, Massachusetts; University of Southern California, Los Angeles, CA; University of Washington, Seattle, WA; University of Washington, Golden, Colorado; UW Medicine Regional Burn Center, Harborview Medical Center, Seattle, WA; UT Southwestern/Parkland Health, Dallas, Texas; Spaulding Rehabilitation Hospital/Harvard Medical School, Boston, MA; UT Southwestern Medical Center, Dallas, Texas; Massachuett General Hospital, Boston, Massachusetts; University of Southern California, Los Angeles, CA; University of Washington, Seattle, WA; University of Washington, Golden, Colorado; UW Medicine Regional Burn Center, Harborview Medical Center, Seattle, WA; UT Southwestern/Parkland Health, Dallas, Texas; Spaulding Rehabilitation Hospital/Harvard Medical School, Boston, MA; UT Southwestern Medical Center, Dallas, Texas; Massachuett General Hospital, Boston, Massachusetts; University of Southern California, Los Angeles, CA; University of Washington, Seattle, WA; University of Washington, Golden, Colorado; UW Medicine Regional Burn Center, Harborview Medical Center, Seattle, WA; UT Southwestern/Parkland Health, Dallas, Texas; Spaulding Rehabilitation Hospital/Harvard Medical School, Boston, MA; UT Southwestern Medical Center, Dallas, Texas; Massachuett General Hospital, Boston, Massachusetts; University of Southern California, Los Angeles, CA; University of Washington, Seattle, WA; University of Washington, Golden, Colorado; UW Medicine Regional Burn Center, Harborview Medical Center, Seattle, WA; UT Southwestern/Parkland Health, Dallas, Texas; Spaulding Rehabilitation Hospital/Harvard Medical School, Boston, MA; UT Southwestern Medical Center, Dallas, Texas; Massachuett General Hospital, Boston, Massachusetts; University of Southern California, Los Angeles, CA; University of Washington, Seattle, WA; University of Washington, Golden, Colorado; UW Medicine Regional Burn Center, Harborview Medical Center, Seattle, WA; UT Southwestern/Parkland Health, Dallas, Texas; Spaulding Rehabilitation Hospital/Harvard Medical School, Boston, MA; UT Southwestern Medical Center, Dallas, Texas; Massachuett General Hospital, Boston, Massachusetts; University of Southern California, Los Angeles, CA

## Abstract

**Introduction:**

Health disparities associated with racial and ethnic minoritized status as well as sex are well documented in burn survivors. Specifically, previous work has found that people from racial and ethnic minority backgrounds as well as females tend to have lower satisfaction with appearance after injury1,2. However, the impact of the intersection between race/ethnicity and sex outside their individual effects has not been well studied. This study aims to assess: 1) the independent association between race/ethnicity and sex with body image, as measured using the Burn Specific Health Scale body image subscale (BSHS-BI), with higher scores corresponding to a better outcome; and 2) test if the association between sex and BSHS-BI differs by survivors’ race/ethnicity.

**Methods:**

Adult participants enrolled in a large longitudinal study who had at least one BSHS-BI score and complete race/ethnicity, sex, age, and total burn surface area (TBSA) data were included. Race/ethnicity and sex were obtained through a combination of self-report and electronic health records. Due to sample size restrictions, race/ethnicity was categorized as White, non-Hispanic; Hispanic; or other, non-Hispanic. A linear mixed effects model was used to analyze the effects of race/ethnicity and sex after adjusting for age, TBSA, and follow-up time. A second linear mixed effects model was used to analyze the interaction between race/ethnicity and sex to determine if race modifies the association between sex and BSHS-BI.

**Results:**

Data from 1,191 participants with injuries from 1996 to 2021 were included in the final analysis. The study sample was796 (66.8%) male, 398 (33.2%) female, 869 (73.0%) White, non-Hispanic, 145 (12.2%) Hispanic, and 177 (14.9%) other, non-Hispanic. The mean age was 43 (standard deviation (SD) = 17) and mean TBSA was 21 (SD = 44). After adjusting for age, TBSA, and follow-up time, mean BSHS-BI score for females was significantly lower than males (0.62 points lower; p < 0.001) and those identifying as Hispanic had mean scores 0.32 (p=.002) higher than White, non-Hispanic and 0.41 (p=0.02) higher than other, non-Hispanic. No significant differences in BSHS-BI were found between White, non-Hispanic and other, non-Hispanic groups. There was no evidence that the association between sex and BSHS-BI differed by race/ethnicity.

**Conclusions:**

Those who self-identified as male and Hispanic had significantly better BSHS-BI scores; however, there was no evidence that the intersection between sex and race/ethnicity was associated with BSHS-BI after accounting for individual sex and race/ethnicity effects.

**Applicability of Research to Practice:**

Consistent with previous research, the results suggest that sex and race/ethnicity are important characteristics to consider when addressing body image changes after burn injury.

